# β Cell-specific deletion of guanylyl cyclase A, the receptor for atrial natriuretic peptide, accelerates obesity-induced glucose intolerance in mice

**DOI:** 10.1186/s12933-018-0747-3

**Published:** 2018-07-17

**Authors:** Sabine Tauscher, Hitoshi Nakagawa, Katharina Völker, Franziska Werner, Lisa Krebes, Tamara Potapenko, Sören Doose, Andreas L. Birkenfeld, Hideo A. Baba, Michaela Kuhn

**Affiliations:** 1 Institute of Physiology, University of Würzburg, Röntgenring 9, 97070 Würzburg, Germany; 2 Department of Biotechnology and Biophysics, University of Würzburg, Am Hubland, 97074 Würzburg, Germany; 3 Paul Langerhans Institute Dresden of the Helmholtz Center Munich at University Hospital and Faculty of Medicine, TU Dresden, Fetscherstraße 74, 01307 Dresden, Germany; 4 Institute of Pathology and Neuropathology, University Hospital Essen, Hufelandstraße 55, 45147 Essen, Germany

**Keywords:** Natriuretic peptides, Guanylyl cyclase-A, Cyclic GMP, β-Cells, Insulin, Obesity

## Abstract

**Background:**

The cardiac hormones atrial (ANP) and B-type natriuretic peptides (BNP) moderate arterial blood pressure and improve energy metabolism as well as insulin sensitivity via their shared cGMP-producing guanylyl cyclase-A (GC-A) receptor. Obesity is associated with impaired NP/GC-A/cGMP signaling, which possibly contributes to the development of type 2 diabetes and its cardiometabolic complications. In vitro, synthetic ANP, via GC-A, stimulates glucose-dependent insulin release from cultured pancreatic islets and β-cell proliferation. However, the relevance for systemic glucose homeostasis in vivo is not known. To dissect whether the endogenous cardiac hormones modulate the secretory function and/or proliferation of β-cells under (patho)physiological conditions in vivo, here we generated a novel genetic mouse model with selective disruption of the GC-A receptor in β-cells.

**Methods:**

Mice with a floxed GC-A gene were bred to Rip-Cre^TG^ mice, thereby deleting GC-A selectively in β-cells (β GC-A KO). Weight gain, glucose tolerance, insulin sensitivity, and glucose-stimulated insulin secretion were monitored in normal diet (ND)- and high-fat diet (HFD)-fed mice. β-cell size and number were measured by immunofluorescence-based islet morphometry.

**Results:**

In vitro, the insulinotropic and proliferative actions of ANP were abolished in islets isolated from β GC-A KO mice. Concordantly, in vivo, infusion of BNP mildly enhanced baseline plasma insulin levels and glucose-induced insulin secretion in control mice. This effect of exogenous BNP was abolished in β GC-A KO mice, corroborating the efficient inactivation of the GC-A receptor in β-cells. Despite this under physiological, ND conditions, fasted and fed insulin levels, glucose-induced insulin secretion, glucose tolerance and β-cell morphology were similar in β GC-A KO mice and control littermates. However, HFD-fed β GC-A KO animals had accelerated glucose intolerance and diminished adaptative β-cell proliferation.

**Conclusions:**

Our studies of β GC-A KO mice demonstrate that the cardiac hormones ANP and BNP do not modulate β-cell’s growth and secretory functions under physiological, normal dietary conditions. However, endogenous NP/GC-A signaling improves the initial adaptative response of β-cells to HFD-induced obesity. Impaired β-cell NP/GC-A signaling in obese individuals might contribute to the development of type 2 diabetes.

## Background

To date, about 40–50% of the US and EU population is overweight and is presenting with a measurable insulin resistance. A significant part of this group will further proceed into development of type 2 diabetes mellitus with increased risk of cardiovascular complications including arterial hypertension and diabetic cardiomyopathy [1]. The heart is not only affected by metabolic disorders, but plays an active role in the defense against them [2–4]. Hence, the two cardiac hormones atrial (ANP) and B-type natriuretic peptides (BNP) not only moderate arterial blood pressure and intravascular volume but additionally improve systemic metabolism [reviewed in 5–7]. These pleiotropic endocrine actions are mediated by their shared transmembrane guanylyl cyclase (GC) receptor, GC-A (also named natriuretic peptide receptor A, NPR-A), which synthesizes cyclic GMP (cGMP) as second messenger [6]. The protective metabolic actions of the NP/GC-A signaling pathway involve lipolytic effects in adipocytes [7–9]; enhanced secretion of the adipokine adiponectin, an insulin-sensitizing hormone; energy expenditure in brown adipose tissue and “browning” of cells in white adipose depots [9]; as well as increased lipid oxidation in skeletal muscle (all reviewed in [5, 6]). Many clinical and experimental studies have demonstrated that obesity is associated with enhanced internalization and degradation of NPs through the NP clearance receptor (NPR-C) on adipocytes, lowering circulating NP levels and endocrine NP/GC-A signaling [10]. Evidence points towards a contribution of this “NP handicap” to the development of type 2 diabetes mellitus and cardiometabolic complications [4–6, 10, 11]. Vice versa genetic variants of the ANP or BNP genes (*NPPA*, *NPPB*) that result in an increase in circulating levels of NPs have been associated not only with lower blood pressure [12], but also with protection from the metabolic syndrome in the general population [13].

Studies with *exogenous*, synthetic NPs suggested that the protective metabolic inter-organ crosstalk effectuated by ANP and BNP might involve the endocrine pancreas. Hence, our previous results with cultured murine β-cells and isolated islets revealed that ANP, via GC-A/cGMP and cGMP-dependent protein kinase I, augments the effects of glucose on K_ATP_ channel activity, Ca^2+^ levels and insulin release [14]. Recently another in vitro study confirmed and extended our observations, showing that ANP enhances the insulinotropic effects of the incretine hormone glucagon like peptide, GLP-1, in isolated murine islets [15]. Interestingly, it was also shown that long-term treatment of cultured pancreatic islets or immortalized β-cell lines with ANP enhances β-cell cyclin-D2 expression and proliferation [16]. The possible relevance for β-cell functions in vivo was addressed by pharmacological studies in human probands. Increased insulin levels and concomitant decreases of plasma glucose were observed during infusion of rather high doses of ANP in healthy subjects [17]. However, in other studies, infusion of more physiological, lower doses of ANP (leading to ~ 2.5-fold increases of plasma ANP levels) either had no effect on plasma insulin levels [18] or elicited only subtle and transient postprandial increases in plasma glucose and insulin [19].

Despite such controversial results, together these pharmacological studies raised the clinical concept that the natriuretic peptide handicap of obese individuals contributes not only to impaired insulin sensitivity but also to inappropriate β-cell insulin secretion [5, 15, 20]. However, all these previous in vivo*/*in vitro studies were performed with *exogenous* administered ANP or BNP, at concentrations which were ~ 100-fold higher as the circulating levels of the endogenous hormones. In fact, no single study addressed whether a NP-mediated axis between the heart and the endocrine pancreas participates in the regulation of β-cells functions under physiological or pathological conditions in vivo. To dissect whether the *endogenous* cardiac NPs regulate insulin secretion and/or the adaptative growth of β-cells, we used *Cre/LoxP* methodology to generate mice with constitutive, β-cell-specific knockout of the GC-A receptor for ANP/BNP (β GC-A KO).

## Methods

### Generation of mice with β-cell-specific inactivation of GC-A

All animal studies were approved by the Animal Care and Use Commitee of Würzburg University and were in accordance with the *Guide for the Care and Use of Laboratory Animals* (NIH Publications No. 8023, revised 1978). To obtain mice with restricted ablation (KO) of GC-A in β-cells, mice with two floxed alleles of GC-A (*GC*-*A*^*fl/fl*^ [21]) were intercrossed with *RipCre*^*tg*^ mice expressing Cre recombinase in β-cells under the control of the rat insulin 2 *promoter*. *RipCre*^*tg*^ mice were a gift from Pablo Herrera, Dept. of Genetic Medicine and Development, University of Geneva, Switzerland [22, 23]. Importantly, all metabolic parameters including β-cell function and morphology in this specific *RipCre*^*tg*^ line are unaltered [23].

Genotypings were performed by PCR of tail tip and tissue DNA using primers GC-A-1 (5′-TCCTGTCTCCCGTGACCTTCC), GC-A-2 (5′-ATCAGAGAATAACCAGCCAGAG) and GC-A-3 (5′-GCATGTAGTTTGTAGTCTCATAC), which amplify a 186-bp fragment for the GC-A (*Npr1*) wild-type allele, a 343-bp fragment for the floxed allele, and a ~ 700 bp fragment for the knockout allele [21]. Presence of the Rip-Cre transgene was detected by PCR using primers Cre1 (5′-GCTGCCACGACCAAGTGACAGCAA) and Cre2 (5′-GTAGTTATTCGGATCATCAGCTACAC) (400-bp band). The hereby generated β GC-A KO mice (*GC*-*A*^*fl/fl*^; *RipCre*^*tg*^) and their littermate controls (*GC*-*A*^*fl/fl*^) were kept under a 12-h light/12-h dark cycle at constant temperature (23 °C) with unlimited access to food and water. All studies were performed with control and KO littermates of mixed background (C57BL/6J and 129SV).

### Metabolic studies

For studies of diet-induced obesity a 60% high-fat diet (HFD, D12492, Research Diets) or the corresponding normal diet with 10% kcal from fat (ND, D12450B, Research Diets) were provided to 5 weeks old male β GC-A KO and control littermates and maintained for 18 weeks [14]. At the defined time points, mice were euthanized under deep (3%) isoflurane anesthesia, and pancreata were processed for histology or dissection of islets.

All in vivo studies were performed between 0800 and 1000 h. Samples for determinations of blood glucose (Accu-check Mobile, Roche Diagnostics, Mannheim, Germany) and plasma insulin (Ultra Sensitive Mouse Insulin ELISA, Crystal Chem, Downers Grove, IL) were obtained from the tail vein [14]. For the oral (oGTT) or intraperitoneal (IP) glucose tolerance tests, mice were previously fasted for ~ 16 h. Mice received 2 g of glucose/kg body weight (BW) in a volume of 10 μl/g BW and blood glucose concentrations were measured at 0, 15, 30, 60, 90 and 120 min [14]. In oGTTs, the samples obtained at 0, 15 and 30 min additionally served to determine basal and glucose-stimulated plasma insulin levels (GSIS). For the insulin tolerance test (ITT), fed mice were starved 4 h before the experiment. Insulin (1 IU/g BW; Actrapid Penfill Insulin human, Novo Nordisk, Bagsværd, Dänemark) was injected intraperitoneally, and blood glucose concentrations were measured at 0, 15, 30 and 60 min. For glucose tolerance (GTT) and sensitivity (ITT), we measured the total area under the curve (AUC). The AUC was calculated from the baseline (0 min) and divided by the period of time [14].

### Determination of arterial blood pressure

Arterial blood pressure levels of mice on ND or HFD were determined by tail-cuff plethysmography [21]. Mice were conditioned by placing them in the holding devices on three consecutive days. Subsequently, blood pressure values per mouse were determined during 15 min each day and averaged for a total of 3 subsequent consecutive days.

### Administration of BNP via osmotic minipumps

Mouse BNP (Bachem) was infused via subcutaneously implanted osmotic minipumps (model 1007D; Alzet Corporation) at a dose of 2 ng/h/g BW over 7 days [9]. The control group received pumps containing saline only.

### Immunohistochemistry and morphometrical analyses of pancreatic sections

Pancreata were fixed with 4% paraformaldehyde, embedded in paraffin and cut in 8-μm sections. Insulin and glucagon stainings were performed with antibodies from Dako (Santa Clara, CA, USA; AB-1001362) and Sigma (Darmstadt, Germany; AB-259852), respectively [14]. Secondary antibodies (Jackson Immuno Research, West Grove, PA, USA) were Alexa Fluor 488 or CY3 coupled and were detected by immunofluorescence. DAPI was used to label nuclei (Dianova). Pictures of three whole sections of the pancreatic corpus per mouse were taken on an Olympus microscope. Fluorescent areas were measured with ImageJ software. The total islet area, area of insulin-positive β-cells and number of β-cells/islet were calculated [14]. The experimentators were blinded regarding genotype and treatment.

### Isolation and incubation of mouse pancreatic islets for determinations of intracellular cGMP, insulin secretion and cyclin-D2 mRNA expression

Pancreatic islets were isolated by collagenase digestion and cultured overnight in RPMI 1640 medium (Gibco) containing 10% FCS [14]. Thereafter, islets were incubated at 37 °C in groups of 35 islets (for cGMP) or 10 islets (for insulin) in a buffer solution containing (in mM): 120 NaCl, 25 NaHCO_3_, 5 KCl, 2.5 CaCl_2_, 1 MgCl_2_, and 3 d-glucose (final pH 7.35). After 2 h, the solution was replaced by fresh solution containing 6 mM d-glucose with or without ANP and the islets were incubated for additional 15 min (for cGMP responses) or 60 min (for insulin secretion). For determination of ANP/cGMP effects, the islets were pretreated with the phosphodiesterase inhibitor 3-isobutyl-1-methylxanthine (0.5 μM IBMX (Sigma), 10 min). cGMP production was stopped by placing islets on ice; after centrifugation (100*g*, 5 min, 4 °C), the islets were lysed with ice-cold 70% (v/v) ethanol. cGMP was determined by radioimmunoassay (RIA) and normalized to sample protein contents (Bradford) [14, 21]. To evaluate the insulinotropic effects of ANP, insulin concentrations in supernatants were determined by ELISA [14]. To study the effects of ANP on mRNA expression levels of cyclin-D2, islets were serum-starved (1% FCS) for 24 h and thereafter ANP was added for another 24 h. The supernatants were discarded and the islets (40 islets per condition) were lysed in TRIzol reagent (Invitrogen).

### Gene expression studies

Total RNA was isolated from cultured or freshly picked mouse islets and cardiac left ventricles using TRIzol reagent (Life Technologies). After reverse transcription (Transcriptor First Strand cDNA Synthesis Kit, Roche), real-time RT-PCR was performed using a LightCycler Instrument (Roche) [24]. The following primers and probes from Roche were used: for GC-A, TGGAGACACAGTCAACACAGC (forward primer); CCGAAGACAAGTGGATCCTG (reverse primer) and probe #71 (cat. no. 04688945001); for BNP, AAG CTG CTG GAG CTG ATA AGA (forward); GTT ACA GCC AAA CGA CTG AC (reverse), with SybrGreen; for cyclin-D2, GCT GTG CAT TTA CAC CGA CA (forward), ACA CTA CCA GTT CCC ACT CCA (reverse) and probe #45 (cat. no. 04688058001). β2 Microglobulin (cat. no. 301208, FAM, Probe 117) or GAPDH (cat. no. 05046211001, Yellow) served as reference genes [24].

### Plasma BNP levels

Plasma BNP levels were measured by enzyme immunoassay according to the manufacturer’s protocol (MyBioSource, Inc., San Diego, CA, USA). BNP was measured instead of ANP because circulating BNP levels showed better correlation with cardiac changes [3].

### Statistical analysis

Data are presented as mean ± SEM (with the number of experiments described in the Legends to Figures). Student’s *t* test or two-way analysis of variance (ANOVA) followed by the Bonferroni post hoc test were used to examine differences between groups, as appropriate. P values < 0.05 were considered statistically significant.

## Results

### β-cell-specific GC-A deletion in *GC*-*A*^*fl/fl*^;*Rip*-*Cre*^*tg*^ mice

**Fig. 1:**
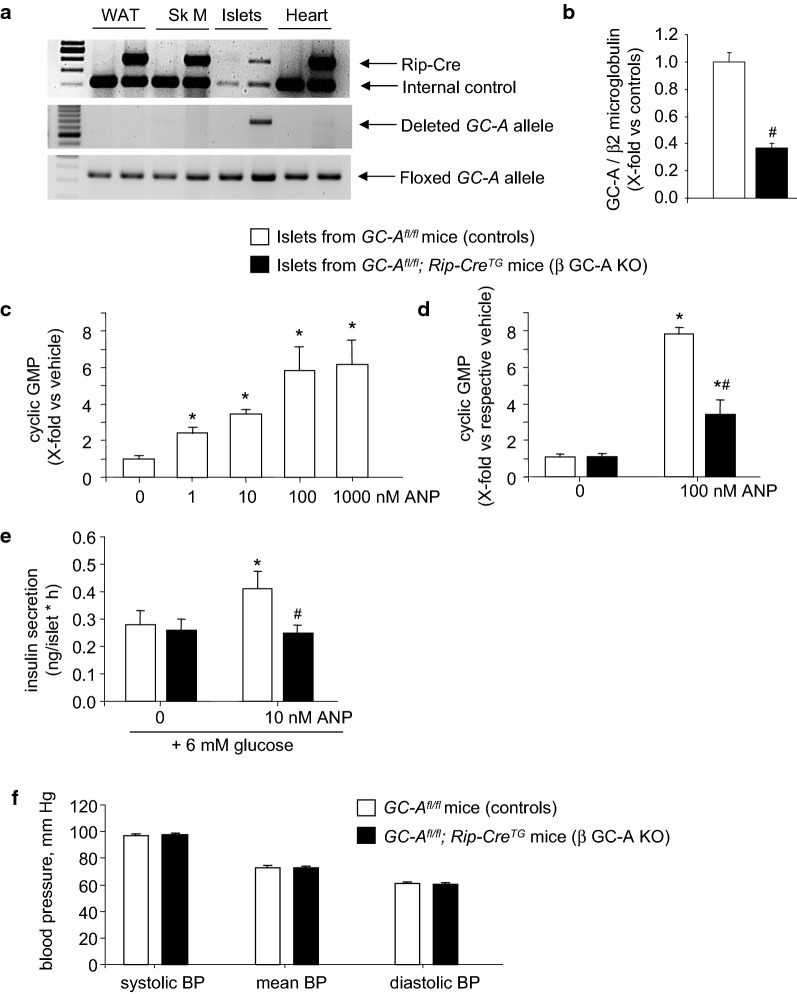
Deletion of GC-A in β-cells from *GC*-*A*^*fl/fl*^*; RipCre*^*tg*^ (β GC-A KO) mice. **a** PCR analysis. Genomic DNA from different tissues was assayed for the appearance of the ~ 700-bp amplicon which results from complete recombination of the floxed GC-A gene segment. Genomic DNA was from white adipose tissue (WAT), skeletal muscle (Sk M), isolated pancreatic islets and hearts. **b** Quantitative RT-PCR analysis. GC-A mRNA expression levels in pancreatic islets from control and β GC-A KO mice. Values are the ratio of GC-A mRNA level relative to β2 microglobulin, expressed as x-fold vs control islets (20 samples per genotype). **c** Cyclic GMP determinations. Concentration-dependent effects of ANP on intracellular cGMP contents of pancreatic islets prepared and cultured from control mice (15 min incubation in the presence of the phosphodiesterase inhibitor IBMX; n = 4 per condition). **d** Comparison of the cGMP responses of β GC-A KO and control islets to 100 nM ANP (15 min incubation in the presence of IBMX; n = 4 per genotype and condition). **e** Insulin release. Effects of ANP on glucose-dependent insulin secretion by pancreatic islets prepared from β GC-A KO and control littermates (1 h incubation; n = 4). **f** Systolic, mean and diastolic arterial blood pressure levels of β GC-A KO and control littermates (n = 16 per genotype). *P < 0.05 vs vehicle; ^#^P < 0.05 vs controls

*GC*-*A*^*fl/fl*^ mice with and without the *Rip*-*Cre*^*tg*^ were born in the expected Mendelian and sex ratios. PCR analysis of genomic DNA demonstrated that Cre-mediated complete recombination of the floxed *GC*-*A* gene only occurred in pancreatic islets (Fig. 1a). No deletion was detected in white adipose tissue, skeletal muscle, heart (Fig. 1a) or other tissues of the doubly transgenic (*GC*-*A*^*fl/fl*^*;Rip*-*Cre*) mice. To characterize the impact of this gene recombination event we studied GC-A mRNA expression and receptor function in islets isolated and cultured from both genotypes, ex vivo. Figure 1b shows that GC-A mRNA expression levels in islets prepared from *GC*-*A*^*fl/fl*^;*Rip*-*Cre*^*tg*^ mice were reduced by ~ 70% (as compared to islets from their *GC*-*A*^*fl/fl*^ littermates). As sensitive assay of ANP/GC-A signaling, we studied cGMP responses of isolated islets to ANP. As shown in Fig. 1c, ANP increased the cGMP contents of control islets (prepared from GC-A^fl/fl^ mice) in a concentration-dependent manner. We then compared the responses of islets from *GC*-*A*^*fl/fl*^ and *GC*-*A*^*fl/fl*^; *Rip*-*Cre*^*tg*^ littermates to a maximal ANP concentration (100 nM). As shown in Fig. 1d, the stimulatory effects of ANP on islets cGMP contents were markedly reduced in islets prepared from the double (*GC*-*A*^*fl/fl*^; *Rip*-*Cre)* transgenic mice as compared to islets from *GC*-*A*^*fl/fl*^ littermates. It is not surprising that the GC-A mRNA expression and cGMP activity were not fully abolished in the islets from the *GC*-*A*^*fl/fl*^; *RipCre*^*tg*^ mice since the GC-A receptor is ablated in β-cells but preserved in other cell types of the islets. In particular, capillary endothelial cells have high GC-A expression levels [6, 24]. To overcome this limitation and dissect specific effects of ANP on β-cell function, we studied islet’s insulin release [14, 15]. As illustrated in Fig. 1e, ANP (10 nM during 1 h) enhanced glucose-dependent insulin secretion in islets isolated from control mice (*GC*-*A*^*fl/f*^) but not in islets obtained from their *GC*-*A*^*fl/fl*^; *RipCre*^*tg*^ littermates. Together, these experiments demonstrate the efficient inactivation of GC-A in β-cells from *GC*-*A*^*fl/fl*^; *RipCre*^*tg*^ mice. Even more, whereas global GC-A KO mice are hypertensive [6], *GC*-*A*^*fl/fl*^; *RipCre*^*tg*^ mice have unaltered arterial blood pressure (Fig. 1f), confirming that apart from β-cells, the effects of ANP/BNP on other target cells are preserved. Hence, we refer such mice as β-cell specific GC-A knockout (β GC-A KO) mice. Their *GC*-*A*^*flox/flox*^ littermates without Cre were used as control mice in all subsequent experiments.

### Disruption of ANP/GC-A signaling in pancreatic β-cells does not alter insulin-glucose homeostasis under physiological, normal dietary conditions

**Fig. 2:**
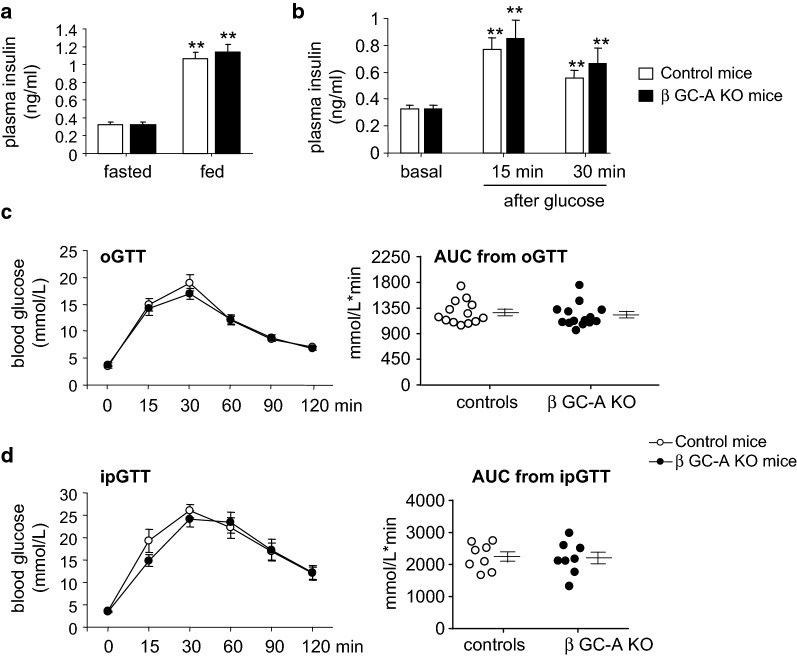
Inactivation of GC-A in pancreatic β-cells (β GC-A KO) does not alter physiological stimulus-secretion coupling in vivo. **a** Fasting (basal) or random fed and **b** oral glucose-stimulated plasma insulin levels (GSIS) were determined in 4 months old GC-A KO and control littermates (n = 13 mice/genotype). **c**, **d** Blood glucose concentration and area under the curve (AUC, right panels) during oral (**c** oGTT (n = 13 per genotype) or IP glucose tolerance tests (**d** ipGTT (n = 8 per genotype). GTTs were performed in 16-h-fasted 4 months old β GC-A KO and control littermates. *P < 0.01 vs fasted or basal

Since *exogenous* ANP enhanced glucose-dependent insulin secretion from control but not from β GC-A KO islets in vitro, we expected that β GC-A KO mice (in vivo) would display diminished insulin levels and impaired glucose tolerance. However, fasting or random fed plasma insulin concentrations and oral glucose-stimulated insulin levels (GSIS) were not different between genotypes (Fig. 2a, b). Consistently, the oGTT and ipGTT revealed that glucose handling was similar in β GC-A KO mice as compared to their control littermates (Fig. 2c, d). Together these studies demonstrate that the endogenous cardiac NP/GC-A signaling pathway does not critically modulate resting or glucose-stimulated secretory functions of β-cells under physiological, normal dietary conditions in vivo.

### Increasing circulating BNP levels by *exogenous* BNP administration enhances glucose-stimulated insulin secretion via the GC-A receptor on β-cells

**Fig. 3:**
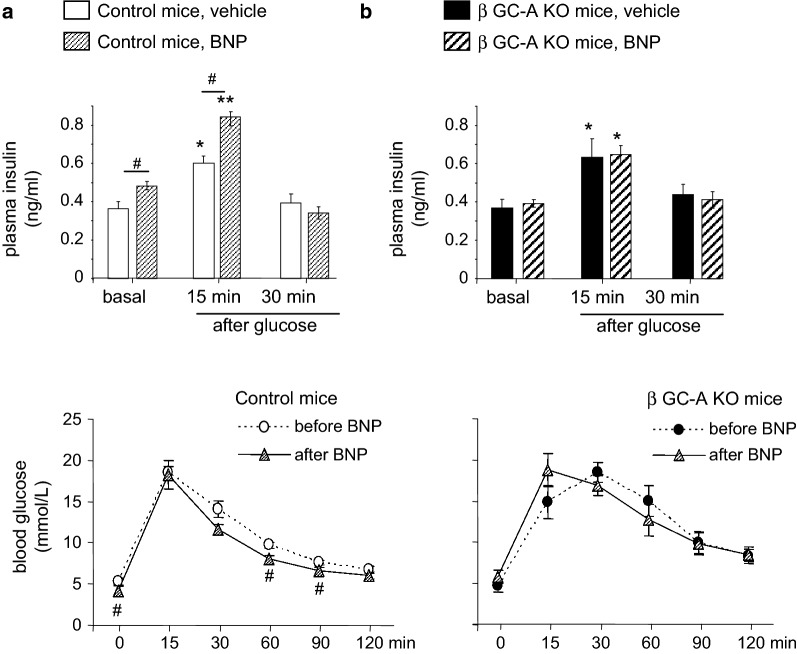
The insulinotropic effects of exogenous BNP are abolished in β GC-A KO mice. Fasted and oral glucose-stimulated plasma insulin levels (GSIS, top) as well as oral glucose tolerance (oGTT, bottom) were determined in 16-h-fasted control (**a**) and β GC-A KO littermates (**b**) after 7 days of subcutaneous vehicle or BNP infusions via osmotic minipumps (n = 6 mice per genotype). *P < 0.05 and **P < 0.01 vs basal; ^#^P < 0.05 vs vehicle treatment (GSIS) or baseline (oGTT before BNP)

Infusion of ANP increases circulating insulin in human probands [17]. To dissect whether this effect is mediated by the GC-A receptor on β-cells, we compared the actions of infused BNP on plasma insulin levels in control and *β GC*-*A KO* mice. We used BNP instead of ANP because it has almost the same affinity for GC-A as ANP, but higher stability and plasma half life [6, 9]. Osmotic minipumps filled with vehicle or synthetic BNP (to deliver 2 ng BNP/h/g BW [9]) were implanted subcutaneously for 7 days. It was previously shown that this protocol results in a 2-fold increase of baseline BNP plasma levels [9]. In the control mice, fasted (basal) and oral glucose-stimulated plasma insulin levels were mildly but significantly enhanced by BNP (Fig. 3a). This was accompanied by a mild decrease in fasted plasma glucose levels and slightly improved oral glucose tolerance (Fig. 3a, bottom). Notably, such effects of infused, *exogenous* BNP on plasma insulin levels and oral glucose tolerance were fully abolished in the *β GC*-*A KO* mice (Fig. 3b). These results extend the observations in isolated islets showing that elevated NP levels can enhance glucose-dependent insulin release. They demonstrate that this effect is mediated by GC-A signaling in β-cells.

### HFD-fed β GC-A KO mice exhibited accelerated glucose intolerance

**Fig. 4:**
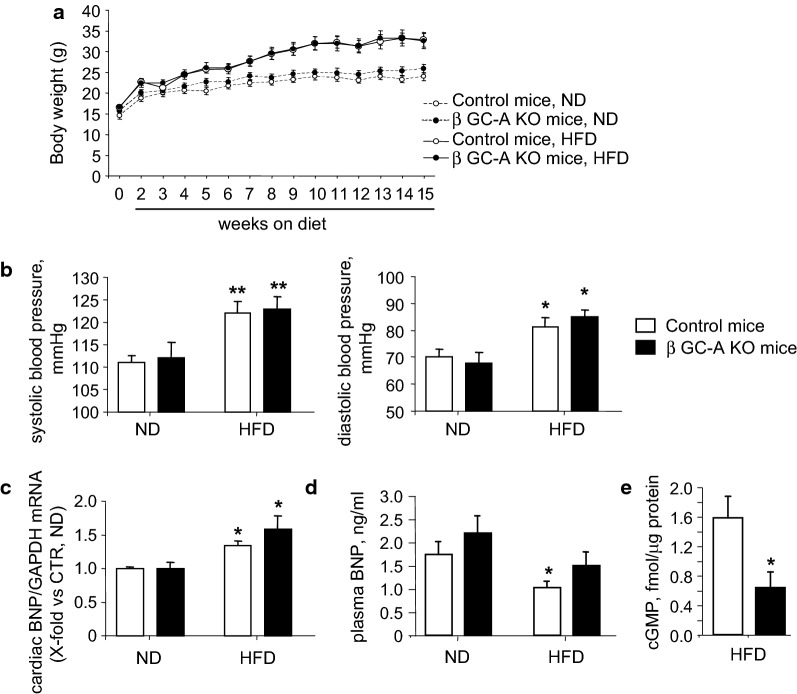
Obesity and arterial hypertension in β GC-A KO and control mice fed a high-fat diet. **a** Body weights of β GC-A KO and control littermate mice before (0 weeks) and during 15 weeks of control (normal, ND) or high-fat diet (HFD). **b** Systolic and diastolic arterial blood pressure levels of β GC-A KO and control mice after 6 weeks of ND or HFD. **c**, **d** Left ventricular BNP mRNA expression and plasma BNP levels after 8 weeks of ND or HFD were determined by real-time RT-PCR and ELISA, respectively. **e** Cyclic GMP contents of islets freshly isolated after 8 weeks of ND or HFD were determined by Radioimmunoassay. n = 7 samples per genotype and condition; *P < 0.05 vs ND

To study whether *endogenous* NP/GC-A signaling improves β-cell functions under pathological conditions such as obesity-induced insulin resistance, control and β GC-A KO mice were fed a HFD starting at 5 weeks of age. Compared to control littermates, β GC-A KO mice had similar body weight gain (Fig. 4a) and food consumption (not shown). After 15 weeks of HFD the mean BWs were 33 ± 1.8 g (control mice) and 32.6 ± 1.8 g (KO mice), representing a weight gain of ~ 23% as compared to mice under ND. In both genotypes, obesity was associated with mild but significant increases of systolic and diastolic blood pressure levels (Fig. 4b). Notably cardiac left ventricular BNP mRNA expression levels were increased under HFD (Fig. 4c). Despite, plasma BNP levels were mildly but significantly decreased under HFD (Fig. 4d). Although the circulating BNP levels were similar in both genotypes (Fig. 4d), the cGMP contents of islets frehsly prepared from the β GC-A KO mice were markedly diminished as compared to controls (Fig. 4e). This corroborates abolished GC-A signaling in β-cells.

**Fig. 5:**
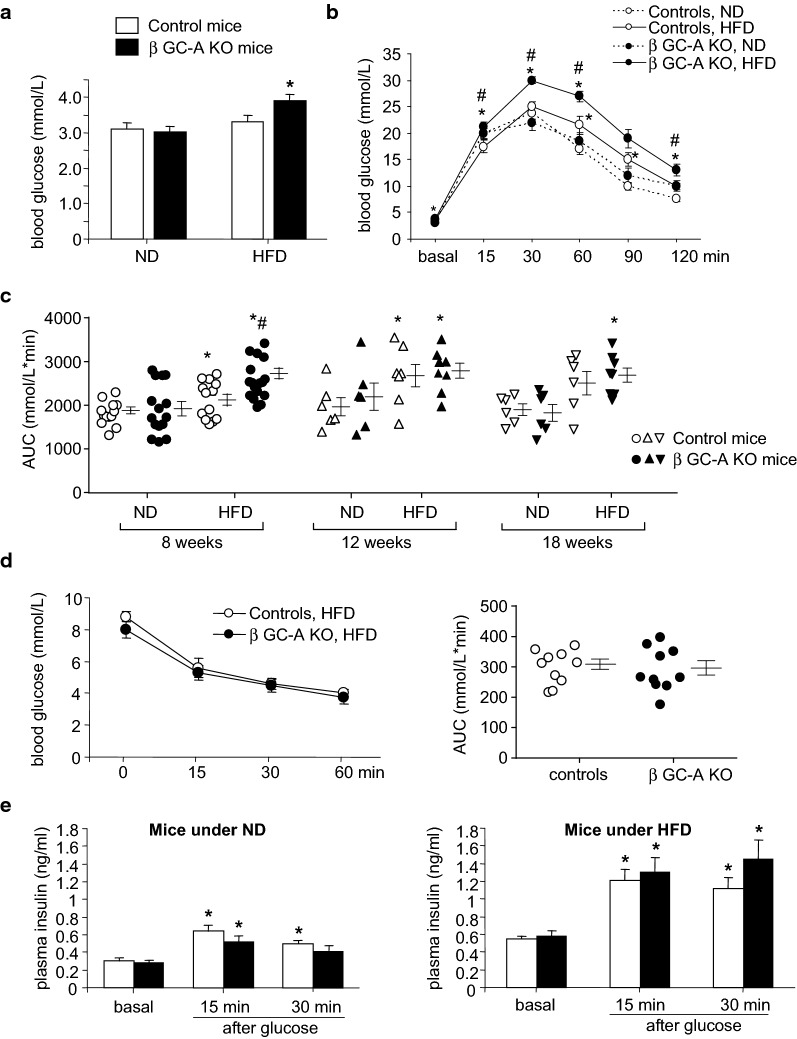
HFD-fed β GC-A KO mice exhibit accelerated glucose intolerance. Fasting blood glucose concentrations (**a**) and blood glucose concentrations during oral glucose tolerance test (oGTT, **b**) were determined in 16-h-fasted β GC-A KO and control littermates after 8 weeks of ND or HFD (n = 13 mice per genotype and condition). **c** Area under the curve (AUC) during oral glucose tolerance tests (oGTT) performed in 16-h-fasted β GC-A KO and control littermates after 8 weeks (n = 13), 12 weeks (n = 6–8) or 18 weeks (n = 6–8) of ND or HFD. **d** Blood glucose concentration and area under the curve (AUC) during insulin tolerance test (ITT) performed in 4-h-fasted β GC-A KO and control mice after 8 weeks of HFD (n = 10 per genotype). **e** Fasting (basal) plasma insulin levels and glucose-stimulated insulin secretion (GSIS) were determined in 16-h-fasted β GC-A KO and control littermates on ND (left panel) or HFD (right panel) (n = 10 per genotype). *P < 0.05 vs ND or basal; ^#^P < 0.05 vs control mice

After 4 weeks on HFD, fasted blood glucose levels and glucose tolerance (oGTT) were still unaltered (not shown). Notably, after 8 weeks on HFD, only the β GC-A KO mice had significantly enhanced fasted glucose levels (Fig. 5a). The oGTT revealed that glucose tolerance was only mildly changed in the control mice but markedly impaired in their KO littermates on HFD (Fig. 5b). Concordantly, the AUC for the oGTT was significantly greater for the β GC-A KO mice compared to control mice on HFD or to KO mice on ND (Fig. 5c). These genotype-dependent differences vanished after 12 and 18 weeks on HFD, with both genotypes now showing similar pathological oGTTs (Fig. 5c). In agreement with a selective abrogation of NP/GC-A signaling in β-cells, the ITT showed similar insulin sensitivities in control and β GC-A KO mice on HFD (Fig. 5d). Figure 5e illustrates that the fasted (basal) and glucose-stimulated plasma insulin levels (GSIS) were markedly higher under HFD (right panel) as compared to ND (left panel), corroborating HFD-induced insulin resistance. However, surprisingly we did not observe genotype-dependent differences.

### The NP/GC-A system improves adaptive β-cell growth in diet-induced obesity

**Fig. 6:**
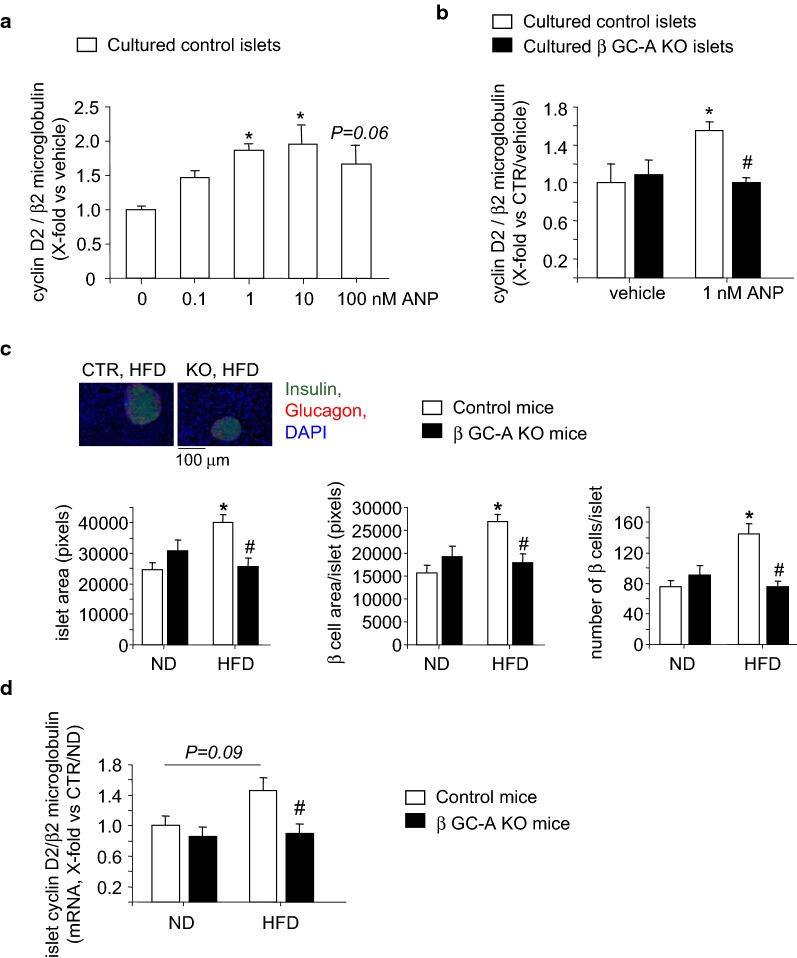
The NP/GC-A system contributes to β-cell proliferation and cyclin-D2 expression in diet-induced insulin obesity. **a**, **b** Effects of ANP on cyclin-D2 mRNA expression in vitro. **a** Concentration–response curve. Pancreatic islets from control mice were cultured in the absence (saline, as vehicle) or presence of different ANP concentrations (0.1–100 nM) for 24 h. **b** Comparison of genotypes. Pancreatic islets from control and β GC-A KO mice were cultured in the absence (saline, as vehicle) or presence of 1 nM ANP for 24 h. Values are the ratio of cyclin-D2 mRNA level relative to β2 microglobulin, determined by quantitative RT-PCR and expressed as x-fold vs vehicle-treated control islets (6–8 samples per condition). **c** Morphometrical analyses of mean islet areas, the area of β-cells per islet and the number of β-cells per islet in pancreatic sections obtained from β GC-A KO and control littermates after 8 weeks of ND or HFD. Top, representative sections stained for insulin, glucagon and cell nuclei (with DAPI) (5 mice per genotype and condition, 3 sections per mouse). **d** Cyclin-D2 mRNA levels relative to β2 microglobulin in islets freshly isolated from β GC-A KO and control littermates after 8 weeks of ND or HFD. Expression levels were determined by quantitative RT-PCR and expressed as x-fold vs control mice receiving an ND (5 mice per genotype and condition, 2 islet sample preparations/mouse). *P < 0.05 vs ND; ^#^P < 0.05 vs control mice

Previous studies showed that ANP enhances β-cell cyclin-D2 expression and proliferation of cultured β-cells in vitro [16]. Accordingly, in the present studies ANP (1–100 nM, 24 h) evoked significant and concentration-dependent increases of cyclin-D2 mRNA expression (as marker for β-cell proliferation) in islets isolated from control mice (Fig. 6a). We then compared the responses of islets from control and *β GC*-*A KO* littermates to the threshold ANP concentration (1 nM). As shown in Fig. 6b, the stimulatory effect of ANP on cyclin-D2 expression was abolished in islets prepared from the KO mice and is therefore mediated by the GC-A receptor.

To follow the hypothesis that the NP/GC-A system contributes to the *initial* adaptative increase in β-cell mass which occurs in clinical and experimental type 2 diabetes [25, 26], we performed studies of islets morphology and gene expression in control and β GC-A KO mice after 8 weeks of HFD or the corresponding ND. Morphometrical analyses of pancreatic sections stained for insulin, glucagon and DAPI demonstrated that in control mice a HFD provoked significant increases of islet areas, i.e. of the area occupied by β-cells, which was due to an increase in their numbers (Fig. 6c). Notably, this adaptative β-cell proliferation was attenuated in β GC-A KO littermates on a HFD (Fig. 6c). Concordantly, quantitative RT-PCR revealed that the expression of cyclin-D2 was enhanced in islets from HFD-fed control mice, although the difference to ND was not statistically significant (P = 0.09; Fig. 6d). Notably, cyclin-D2 levels in islets prepared from HFD-fed β GC-A KO mice were clearly and significantly lower in comparison to islets from HFD-fed control littermates (Fig. 6d). Together these observations indicate that NPs support adaptative β-cell proliferation during early stages of increased insulin demand, here provoked by HFD as experimental model of type 2 diabetes.

## Discussion

Cardiac natriuretic peptides are well known for their cardiovascular functions [6]. In the past years, many published experimental and clinical investigations demonstrated that NPs, via the GC-A receptor, improve insulin sensitivity in the adipose tissue, skeletal muscle, and liver, thereby involving the heart in energy metabolism [2, 5–9]. Recent studies of isolated cultured mouse pancreatic islets indicated that NP/GC-A signaling may additionally enhance stimulus-secretion coupling of pancreatic β-cells: directly, by augmenting glucose-dependent insulin release; and indirectly, by potentiating the effect of the incretin hormone GLP-1 [14, 15]. Such in vitro studies raised the concept that NPs connect the heart with the endocrine pancreas to enhance physiological postprandial insulin secretion [15, 20, 27]. However, they harbor the important limitation that the synthetic NPs were tested at concentrations which were > 100 to 1000-folds higher (10 nM) as compared to the physiological range of resting circulating ANP or BNP levels in healthy probands (10–50 pM [28–30]) or mice (100–200 pM [31]; ~ 300 pM BNP in the present study). Even after a meal or a 75-g oGTT, the plasma concentrations of ANP in humans only increase modestly by ~ 10% [28]. Larger, 2 to 10-fold increases of plasma ANP/BNP levels are induced by exercise [29] or exposure to cold temperatures [9]. Because the concept of an NP-mediated endocrine connection between the heart and the pancreas would have important (patho)physiological and clinical implications, the present in vivo studies addressed three questions: (1) does NP/GC-A signaling in β-cells modulate baseline or *acute* glucose-stimulated insulin secretion under resting, physiological conditions in vivo?; (2) does a subtle, more physiological increase of plasma NP levels, as occurs for instance in exercise or coldness [9, 29], improve glucose-stimulated insulin release?; and (3) does impaired NP/GC-A signaling in β-cells enhance their vulnerability to diabetogenic stimuli, such as HFD? To answer these questions we performed metabolic studies (in vivo) in mice with selective inactivation of the GC-A receptor in β-cells.

### Does NP/GC-A signaling in β-cells modulate stimulus-secretion coupling under physiological conditions?

As illustrated, ANP enhanced glucose-dependent insulin release from control but not from β GC-A KO islets, which is consistant with other published in vitro studies [14, 15]. However, under baseline, physiological conditions glucose-induced insulin secretion (GSIS) and oral or IP glucose tolerance were not different between β GC-A KO and control littermates (in vivo). The fact that oral and IP glucose had similar effects on plasma glucose and insulin levels in both genotypes largely excludes the possibility that the *endogenous* cardiac NPs, at physiological plasma levels, augment stimulus-secretion coupling or the modulatory effects of incretines such as GLP-1 in vivo, for instance after a meal. Also, β-cell size and numbers were not different between the two genotypes. Hence, although β-cells express GC-A receptors [14–16; present study], NP/GC-A signaling is dispensable for baseline or postprandial insulin release under resting, physiological conditions.

### Does a mild chronic increase of plasma NP levels improve β-cell insulin secretion?

In our pharmacological studies, a low dose of BNP infused during 7 days, resulting in a ~ 2-fold increase of plasma BNP levels [9], mildly but significantly increased baseline and glucose-stimulated insulin levels and improved oral glucose tolerance in control mice. These effects of *exogenous* BNP were abolished in β GC-A KO littermates demonstrating that they were mediated by β-cell GC-A signaling. Notably human studies have shown an association of a single nucleotide polymorphism rs5068 on the natriuretic peptide precursor A (*NPPA*) locus gene with higher levels of circulating ANP and lower likelihood of incident diabetes [13]. Our data indicate that this inverse correlation might be partly due to protective effects of sustained elevations of *endogenous* NP on β-cell functions.

### Does impaired NP/GC-A signaling in β-cells enhance their vulnerability to diabetogenic stimuli, such as HFD?

In general, apart from their diverse *acute* cardiovascular and metabolic effects, NPs exert *chronic* effects on cell growth and proliferation. They inhibit cardiac myocyte hypertrophy and fibroblast proliferation, but stimulate the proliferation of vascular endothelial cells or renal podocytes (all reviewed in [6]). These studies also demonstrated that such effects of NPs are not relevant under baseline, physiological conditions but markedly impact cardiac remodeling, angiogenesis or podocyte regeneration under disease conditions [32–34]. As already mentioned, it was also shown that ANP can stimulate the proliferation of cultured rat pancreatic islets β-cells and insulinoma cells (INS-1E) via GC-A/cGMP-dependent activation of phosphatidylinositol 3′-kinase and Akt/Foxo1a/cyclin-D2 signaling [16]. In line with these observations, ANP enhanced cyclin-D2 mRNA expression levels in islets isolated from control but not from *β GC*-*A KO* mice (present studies). It is well known that under physiological conditions, β-cells barely proliferate. However, in initial stages of clinical or experimental prediabetes type 2, e.g. in obesity, the endocrine pancreas compensates for insulin resistance by increasing β-cell mass and insulin secretion [25, 26, 35]. Later on β-cell failure caused by a loss of cells and reduced insulin secretion from remaining individual β-cells leads to overt diabetes [35]. To study whether in this situation the *endogenous* NP/GC-A system modulates β-cell proliferation and function, control and β GC-A KO littermates were fed a HFD, a condition known to provoke obesity, arterial hypertension, insulin resistance and enhanced β-cell proliferation in mice [8, 35, 36]. Control and β GC-A KO mice showed similar weight gain, insulin sensitivity (ITT) and arterial hypertension under HFD. Cardiac BNP mRNA expression levels were doubled, possibly in response to increased cardiac afterload [3]. However, circulating BNP levels were lower under HFD as compared to ND, which is consistant with many published clinical and experimental studies showing that obese and type-2 diabetics display reduced circulating NP levels [5]. This has been linked to enhanced expression of the natriuretic peptide clearance receptor, NPR-C, in adipocytes [5, 9, 37]. Importantly, while these systemic effects of HFD (on blood pressure and BNP expression and circulation) were similar in control and β GC-A KO mice, islets freshly prepared from the later mice had markedly reduced cGMP contents, which is consistant with local ablation of the GC-A receptor.

In control mice, the size of the endocrine islets, the total number of β-cells per islet as well as islet cyclin-D2 expression levels were already increased after 2 months of high-fat feeding. Notably, these adaptative morphological and molecular changes were absent in β GC-A KO mice. Accordingly, these mice showed accelerated glucose intolerance already at 2 months of HFD (shown by oGTT), although plasma insulin levels apparently were unaltered. The mismatch between the increased glucose excursion during the oGTT in β GC-A KO mice and similar glucose-induced insulin levels (GSIS) in comparison to control littermates suggests that islet β-cells lacking GC-A are not able to compensate sufficiently for an increased glucose excursion during the development of an increased insulin demand (here provoked by HFD). Further studies are needed to clarify the exact mechanism.

The molecular pathways that are activated and drive the increases in β-cell mass in obesity and insulin resistance have yet to be fully elucidated [38]. High glucose levels and incretine hormones such as GLP-1 might be involved [38, 39]. Our data suggest that cardiac NPs, via GC-A/cGMP signaling, support these pathways and thereby the adaptative proliferation of β-cells during early stages of obesity-linked insulin resistance and metabolic syndrome.

### Limitations of the study

Our study harbors three limitations:

- We could not corroborate β-cell GC-A deletion by immunohistochemistry. We have tested commercially available and own anti-GC-A antibodies and observed unspecific cross-reactions of all antibodies. However, the following functional experiments demonstrated the efficient deletion of GC-A in β-cells from the *GC*-*A*^*fl/fl*^*; RipCre*^*tg*^ mice: the stimulatory effects of synthetic NPs on glucose-dependent insulin release (in vitro*/*in vivo) and islet cyclin-D2 expression (in vitro) were fully abolished. Corroborating these results, a recently published in vitro study showed that synthetic ANP modulates K_ATP_ channel activity and Ca^2+^ levels of β-cells isolated from the here generated *GC*-*A*^*fl/fl*^ mice but not from their *GC*-*A*^*fl/fl*^; *RipCre*^*tg*^ littermates [15]. In addition, we have demonstrated the efficient Cre/lox-mediated cell-restricted deletion of GC-A in many previous studies with these *GC*-*A*^*fl/fl*^ mice [21, 32–34].
- Transcription from the Rip (insulin 2) promoter starts during pancreas development [23]. We cannot exclude adaptative developmental changes in the double transgenic (*GC*-*A*^*fl/fl*^; *RipCre*^*tg*^) mice, but the absence of changes in β-cell and islet morphology and function under baseline (normal dietary) conditions throughout age argues against developmental changes.
- Throughout all experiments, we compared *GC*-*A*^*fl/fl*^ (controls) and their β GC-A KO littermates. Rip-Cre mice were not included as a control group, since (i) they cannot be studied as littermates; (ii) we did not observe genotype-dependent differences in oGTT, insulin release and sensitivity or body weights at most time points; and (iii) previous studies demonstrated that metabolic parameters and β-cell function and morphology in this specific Rip-Cre line are undistinguishable from control mice [23].

## Conclusions

Our comparative investigations of β GC-A KO and control littermates reveal that the cardiac hormones ANP and BNP do not modulate stimulus-secretion coupling of pancreatic β-cells under resting, physiological conditions. However, endogenous NPs contribute to the control of glucose homeostasis under conditions of pathological diet-induced obesity by improving β-cell proliferation and function in early stages of enhanced insulin demand. Large prospective studies in nondiabetic individuals showed that low initial plasma levels of ANP or BNP predict development of future diabetes and glucose progression over time, suggesting a causal role of chronic NP deficiency in diabetes development [11, 37, 40]. Conversely, higher NT-proBNP is associated with decreased risk of incident diabetes even after adjustment for traditional risk factors [41]. Our experimental observations add an important novel piece of information, indicating that deficient NP/GC-A signaling in β-cells impairs adaptative β-cell proliferation and glucose tolerance during developing obesity, which could be involved in the pathogenesis of type 2 diabetes in its early stages.

## Abbreviations

ANP: atrial natriuretic peptide
BNP: B-type natriuretic peptide
GC-A: guanylyl cyclase A (also named NPR-A: natriuretic peptide receptor type A)
β GC-A KO: mice with β-cell restricted deletion of the GC-A receptor
oGTT: oral glucose tolerance test
ITT: insulin tolerance test
GSIS: glucose stimulated insulin secretion
